# Interatrial Shunt Device for Heart Failure With Preserved Ejection Fraction

**DOI:** 10.3389/fcvm.2019.00143

**Published:** 2019-09-18

**Authors:** David M. Kaye, Shane Nanayakkara

**Affiliations:** Heart Failure Research Group, Department of Cardiology, Baker Heart and Diabetes Institute, Alfred Hospital, Melbourne, VIC, Australia

**Keywords:** heart failure, diastolic dysfunction, structural heart device, clinical trial, cardiac catheter interventional treatment

## Abstract

Heart failure with preserved ejection fraction (HFpEF) accounts for approximately half of the current burden of HF, and the prevalence is continuing to rise. In contrast to HF with reduced ejection fraction (HFrEF) there are no clinically effective evidence based therapies for HFpEF. The principal pathophysiologic disorder is an elevation of left atrial pressure, most notable during physical activity, which results from impaired left ventricular diastolic reserve, and increased left atrial stiffness. This review outlines the clinical development of a potential device based therapy for HFpEF, the interatrial shunt device (IASD).

Heart failure (HF) is one of the most prevalent cardiovascular disorders and recent data highlight a continuing rise in the number of people living with HF, most notably in the context of an aging population (1). In particular, incident HF with preserved ejection fraction (HFpEF), defined by a left ventricular ejection fraction of >50%, has emerged over recent years to be at least equal in importance to that of HF with reduced ejection fraction (HFrEF) (2). In older persons, particularly in women, HFpEF is rapidly emerging as the most prevalent form of HF (3). This epidemiologic transition, reflects the rising prevalence both non-modifiable and modifiable risk factors such as aging, hypertension, diabetes, and obesity (2, 4–6). The increased diagnosis also reflects increased clinical focus on HFpEF and the availability of diagnostic tools, such as echocardiography and natriuretic peptide assays. The functional limitation and reduction in quality of life experienced by HFpEF patients is similar to that in patients with HFrEF (7, 8). Overall survival in HFpEF patients is worse than that in a healthy age-matched population (9–12) and in several studies similar to that of HFrEF. Currently, HFpEF treatment represents one of the most challenging problems in cardiovascular medicine, given that to date, pharmacological treatments with proven effectiveness in HFrEF, have failed to demonstrate benefit in large scale clinical trials (13–22).

As outlined in Figure 1, the pathophysiologic basis of HFpEF is complex and the clinical features represent the integrated effect of a range of abnormalities in cardiac, vascular and non-cardiovascular comorbidities. Elevated left atrial (LA) pressure is considered to be the hallmark feature of HFpEF, often only becoming apparent during physical activity (23–25). We previously showed that although the resting pulmonary capillary wedge pressure (PCWP) tends to be greater in HFpEF patients than in healthy control subjects and patients classified with non-cardiac dyspnea (NCD) (26, 27), the PCWP is often within the proscribed “normal” range. However, in contrast to normal subjects or NCD patients, individuals with HFpEF display a characteristic marked rise in PCWP at low workload (Figure 2). The magnitude of the rise in PCWP during physical activity has been demonstrated to correlate with functional capacity and with outcomes (25, 28).

**Figure 1:**
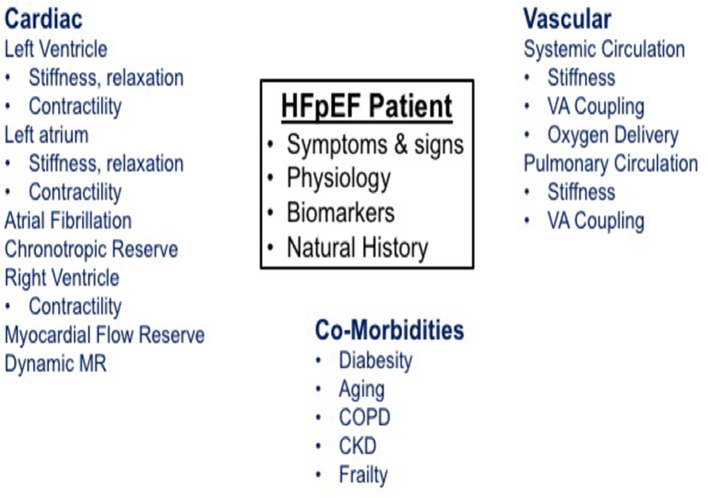
Schematic represents the pathophysiology of heart failure with preserved ejection fraction.

**Figure 2:**
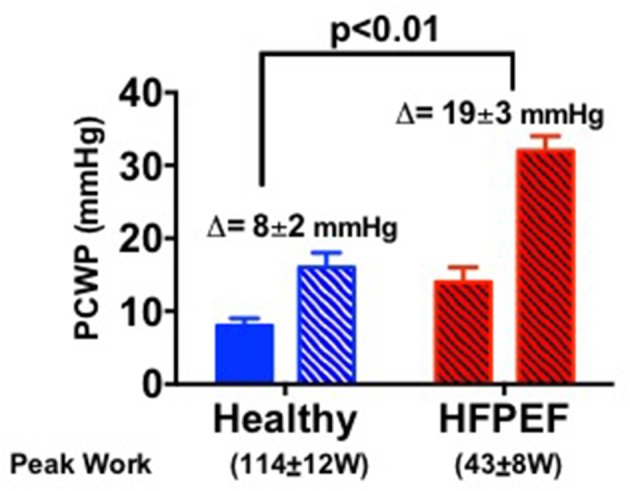
Bar graphs show the pulmonary capillary wedge pressure (PCWP) response to exercise in healthy people and in HFpEF patients [adapted from van Empel et al. (26)].

Various mechanisms contribute to the disproportionate, exercise mediated rise in LA pressure in HFpEF including increased left ventricular and LA stiffness together with impaired active relaxation (27, 29, 30). The cause of abnormal ventricular diastolic and atrial mechanics is complicated. Although left ventricular hypertrophy has been incorporated into diagnostic algorithms for HFpEF (6), its present is not at all uniform in HFpEF (31). Myocardial fibrosis is commonly observed in HFpEF (32) and likely contributes substantially to increased ventricular and atrial stiffness. However, the capacity pharmacological interventions to substantially reverse fibrosis appears to be limited, at least in the short to intermediate term (33). Aside from fibrosis as therapeutic target in HFpEF, pharmacologic interventions directed toward pathways that influence diastolic performance by improved myocardial blood flow, active relaxation, or cardiomyocyte stiffness have also been studied. In particular, a substantial range of clinical studies of agents directed toward the nitric oxide/cyclic guanosine monophosphate/protein kinase G pathway have been conducted (17, 18, 21, 22, 34), however none have yielded clear benefit.

In the absence of effective drug therapies for HFpEF, a device-based approach to reduce LA pressure, especially in the context of the exertional rise, has been developed. This principle was based upon the observation that patients the combination of mitral stenosis and a congenital atrial septal defect (Lutembacher's syndrome) are less symptomatic than patients with isolated mitral stenosis of similar severity due to LA pressure offloading. Based upon invasive hemodynamic data obtained from patients with symptomatic HF and an LVEF ≥ 40%, computer modeling studies were conducted to determine the optimal size of an interatrial shunt that would result in a lower peak LA pressure, whilst avoiding excessive left to right shunting (35). As shown in Figure 3, simulation studies identified that an 8 mm interatrial shunt would substantially attenuate the activity based rise in LA pressure, whilst creating only a small resting left to right shunt of 1.3–1.4:1.

**Figure 3:**
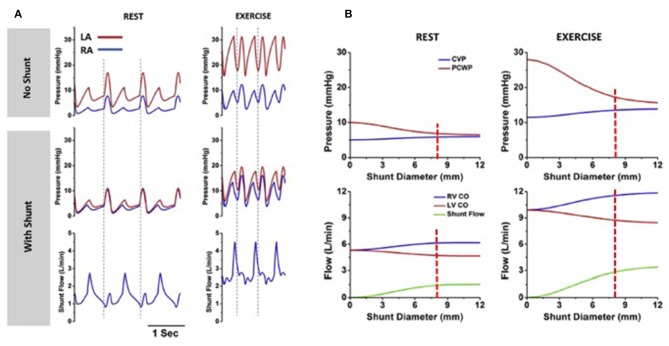
Computer simulations, based on clinical data, show rest and exercise left and right atrial pressures in the absence and presence of the interatrial shunt device (left panel). Right panel shows the influence of shunt diameter on the pressure reduction and amount of left to right shunting (Permission for re-use obtained from Elsevier). **(A)** Corvia IASD device *ex vivo*. **(B)** IASD device *in vivo* (porcine heart).

Based on these computations an interatrial shunt device (IASD, Corvia Medical, Tewksbury, MA, USA) was developed to be positioned within the atrial septum and to provide continuous left to right shunting through an 8 mm central lumen (Figure 4). The implant procedure requires trans-septal catheterization and the passage of a 16Fr delivery sheath. The trans-septal puncture is performed using standard techniques, supported by either transesophageal or intra-cardiac echocardiography. The delivery catheter is advanced over a guide wire into the LA, followed by deployment of the left side of the IASD (Figure 4C). Subsequently, the delivery system is retracted to cause the opened arms of the IASD to abut the inter-atrial septum on the left atrial side of the septum. Following confirmation of apposition to the septum, the right side of the device is deployed, thereby locating the IASD in position. IASD patients are treated with lifelong aspirin together with short-term (typically 3–6 months) clopidogrel. Experience with other similar devices such as the “V-Wave” system has also been reported (36).

**Figure 4:**
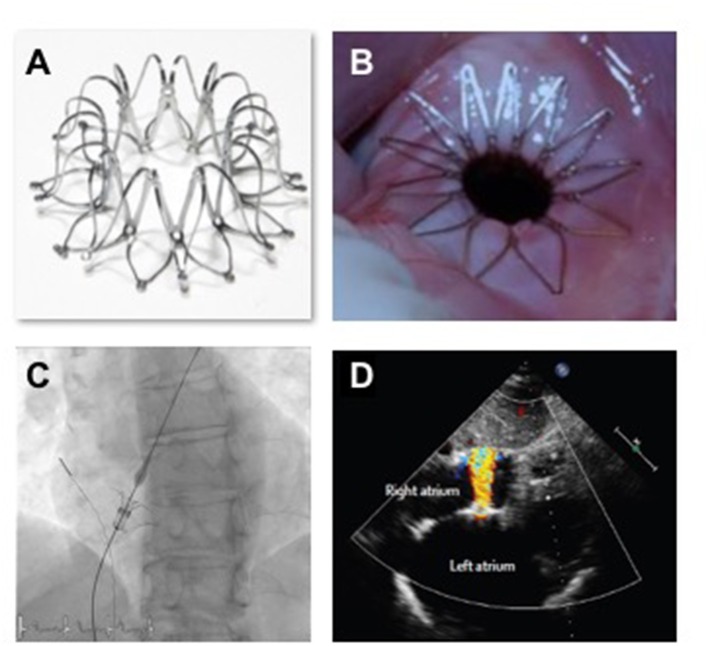
Images **(A,B)** show the IASD; **(C)** show the fluoroscopic image of IASD delivery; **(D)** echocardiographic image showing left to right blood flow.

Following an initial pilot study of the IASD which confirmed safety together with evidence of improved hemodynamics (37) a multi-center, open-label, non-randomized study of the IASD was conducted with clinical, echocardiographic, and hemodynamic follow-up after 6 months and 1 year (38, 39). Patients with known HFpEF were included if they had evidence of chronic symptomatic HF (NYHA class II-IV), a left ventricular ejection fraction >40% and an elevated PCWP at rest (>15 mmHg) or during exercise (>25 mmHg) measured by right heart catheterization. Patients with significant right ventricular dysfunction including a central venous pressure (CVP) >14 mmHg and tricuspid annular plane systolic excursion (TAPSE) <14 mm were excluded to reduce the potential risk of right heart failure and to ensure that left atrial pressure exceeded right atrial pressure to ensure continued left-to-right interatrial shunting. The IASD was successfully implanted in 64 patients and there were no device related complications. At 6 months, in this open label study, there was evidence of significant improvement in quality of life measures and functional status and evidence of an improvement in the peak exercise PCWP especially when corrected for workload (38). Oximetric assessment of the left to right shunt revealed a Qp:Qs of ~1g:1, consistent with the prior modeling work. IASD implantation was associated with a modest but significant increase in right atrial pressure, right atrial volume, and right ventricular volume together with an increase in right sided cardiac output (38). At the 1 year follow up there was evidence of sustained clinical benefit with regards to quality of life, functional capacity and the hemodynamic profile whilst the echocardiographic features of right sided remodeling were stable (39). Evidence of hemodynamic benefit, with a reduction in PCWP with the IASD during exercise has recently been confirmed in a small randomized, parallel group, blinded study (40).

These encouraging data have prompted the commencement of a pivotal multi-center randomized clinical trial (REDUCE LAP HF-II, NCT03088033) of 608 patients, with hemodynamically confirmed HFpEF, randomized to IASD implant, or sham procedure and followed for 5 years. Cross over will be allowed at 24 months. The primary endpoint of the study is a composite of the incidence of cardiovascular mortality or ischemic stroke, the rate of heart failure admissions or healthcare facility visits for IV diuresis, and a change in the KCCQ total summary score. In this context, it is relevant to consider the nature of overall and cardiovascular mortality in patients with HFpEF. Epidemiologic studies highlight the complex, multimorbid nature of HFpEF patients in which mortality relates predominantly to cardiovascular events, however with a significant contribution from non-cardiovascular events (12).

An alternate device, the V-Wave System (V-Wave, Caesarea, Israel) has also been trialed in HF. The device is an hourglass shaped self-expanding prosthesis on a nitinol frame, with a polytetrafluoroethylene (PTFE) skirt extending across both sides of the septum. The central lumen is smaller than the Corvia device (5.1 mm), and includes a porcine trileaflet valve on the right atrial side designed to prevent right-to-left flow. All patients were anticoagulated for 3 months post implantation. Thirty-eight patients were enrolled in a single arm feasibility study, with 100% initial implant success. All forms of heart failure were included, with 8 (21%) having an ejection fraction over 40%. Half of the devices were either occluded or stenotic by 12 months, with some evidence of improved hemodynamics in those with patent devices. Further randomized data in the HFrEF and HFpEF population are required following further device modification to improve shunt patency.

The precise nature and type of cardiovascular mortality in HFpEF is not well-characterized, given the limitations of available datasets, however it appears to result from a combination of heart failure, MI, sudden death, and stroke. The potential effect of IASD implantation on HF outcomes has been investigated recently by modeling the predicted outcome of the REDUCE LAP-HF cohort in the absence of the IASD using the MAGGIC prognostic model (41, 42), and comparing it to actual outcomes over a 3 years follow-up period (43). Although this study was small in size and there were relatively few death, it was shown that the observed mortality was significantly lower than that predicted. Further *post-hoc* analysis in this study also demonstrated that patients who had a particularly favorable hemodynamic response to IASD implantation had lower rates of HF hospitalization. The potential for IASD implantation to impact on survival is consistent with the study by Dorfs et al. who showed that a higher workload correct exercise PCWP was associated with poorer survival (28).

Given the complex nature of HFpEF and the invasive nature of IASD implantation, as compared to pharmacotherapy, it is appropriate to consider those patients who might benefit most from the procedure. Given that the IASD implant functions to reduce LA pressure, attempts have been made to better understand the factors that contribute most to LA pressure particularly during exercise. Wessler et al. demonstrated that the magnitude of the reduction in exercise PCWP is influenced by the baseline pressure gradient between left and right atria (44). As such, the pressure of right heart failure and elevated right atrial pressure is of particular concern in patients being considered for IASD implantation. In conjunction, it may also possible to identify patients that may derive the greatest benefit by predict those that would have the greatest elevation of exercise LA pressure. Telles et al. have recently shown that patients with more marked reductions in LA strain appear to have the greatest increase in exercise PCWP and therefore might derive greater benefit (27). An increased rate of atrial fibrillation is also a potential consequence of a device inserted in the atrial septum; conversely, a reduction in left atrial pressure may also reduce the risk of AF.

Taken together, the management of HFpEF patients continues to be a major, unresolved clinical challenge. Despite the complexity of the pathophysiological pathways leading to the development of HFpEF, the fundamental final consequence is an elevation in left atrial pressure. The IASD was developed to specifically address this physiologic target and early studies have shown the device to be safe and with promising evidence of efficacy. A pivotal randomized controlled study, REDUCE LAP-HF II is currently underway to definitely determine the clinical utility of this procedure.

## Author Contributions

All authors listed have made a substantial, direct and intellectual contribution to the work, and approved it for publication.

### Conflict of Interest Statement

The authors declare that the research was conducted in the absence of any commercial or financial relationships that could be construed as a potential conflict of interest.
